# Quasi-elastic neutron scattering reveals the relationship between the dynamical behavior of phospholipid headgroups and hydration water

**DOI:** 10.1063/4.0000184

**Published:** 2023-08-21

**Authors:** Md. Khalidur Rahman, Takeshi Yamada, Norifumi L. Yamada, Mafumi Hishida, Yuji Higuchi, Hideki Seto

**Affiliations:** 1 Department of Materials Structure Science, The Graduate University for Advanced Studies (SOKENDAI), 1-1 Oho, Tsukuba, Ibaraki 305-0801, Japan; 2 Institute of Materials Structure Science/J-PARC Center, High Energy Accelerator Research Organization (KEK), Tokai, Naka, Ibaraki 319-1106, Japan; 3 Neutron Science and Technology Center, Comprehensive Research Organization for Science and Society (CROSS), Tokai, Naka, Ibaraki 319-1106, Japan; 4 Department of Chemistry, Faculty of Pure and Applied Sciences, University of Tsukuba, Tsukuba, Ibaraki 305-8571, Japan; 5 Research Institute for Information Technology, Kyushu University, 744 Motooka, Nishi-Ku, Fukuoka 819-0395, Japan

## Abstract

The dynamics of hydration water (HW) in 1,2-dimyristoyl-*sn*-glycero-3-phosphoethanolamine (DMPE) was investigated by means of quasi-elastic neutron scattering (QENS) and compared with those observed in 1,2-dimyristoyl-*sn*-glycero-3-phosphocholine (DMPC). The headgroup dynamics of DMPE was investigated using a mixture of tail-deuterated DMPE and D_2_O, and the QENS profiles were interpreted as consisting of three modes. The fast mode comprised the rotation of hydrogen atoms in –NH_3_^+^ and –CH_2_– groups in the headgroup of DMPE, the medium-speed mode comprised fluctuations in the entire DMPE molecule, and the slow mode comprised fluctuations in the membrane. These interpretations were confirmed using molecular dynamics (MD) simulations. The HW dynamics analysis was performed on a tail-deuterated DMPE and H_2_O mixture. The QENS profiles were analyzed in terms of three modes: (1) a slow mode, identified as loosely bound HW in the DMPC membrane; (2) a medium-speed mode similar to free HW in the DMPC membrane; and (3) a fast mode, identified as rotational motion. The relaxation time for the fast mode was approximately six times shorter than that of rotational water in DMPC, consistent with the results of terahertz time-domain spectroscopy. The activation energy of medium-speed HW in DMPE differed from that of free HW in DMPC, suggesting the presence of different hydration states or hydrogen-bonded networks around the phosphocholine and phosphoethanolamine headgroups.

## I. INTRODUCTION

Biological functions dependent on the hydration state of active biomolecules are related to water dynamics, for example, protein dynamics.^1^ In a drug-treated cell,^2^ the dynamics of water in the cytoplasm slows down. Therefore, understanding the structure and dynamics of water molecules surrounding biomolecules is essential. Recently, the water dynamics of phospholipid bilayers acting as biomembrane models has attracted increasing attention.^3^

Water molecule interacts with the hydrophilic portion of a phospholipid, termed HW, which behaves differently compared with bulk water. Various researchers have studied HW in the vicinity of lipid membranes and investigated its properties using a range of experimental techniques and simulation tools. Aoki and Kodama conducted differential scanning calorimetry (DSC) and revealed the existence of non-freezable, freezable, and bulk water upon the addition of up to 32 g% of water to DMPE.^4^ Non-freezable water is considered tightly bound water directly connected to the DMPE headgroup, and freezable water is considered loosely bound water weakly connected to the DMPE headgroup. Zhao *et al.* observed the dynamics of water in a stack of bilayers of the phospholipid 1,2-dilauroyl-*sn*-glycero-3-phosphocholine (DLPC) using ultra-fast polarization selective vibrational pump–probe spectroscopy.^5^ They explained how water dynamics near the hydrophobic choline group differs from those near the phosphate group.^5^ Marrink *et al.* investigated water between 1,2-dipalmitoyl-*sn*-glycero-3-phosphocholine (DPPC) bilayers using MD simulations and showed hydrogen bonding and diffusion of water.^6^ They concluded that the structure of water is perturbed between the DPPC bilayers at the liquid crystalline phase, and that the hydration force depends on the water structure and propagation of DPPC.^6^ Lechner *et al.* investigated a purple membrane multilamellar system as a function of temperature using neutron diffraction. Their investigation revealed that the purple membrane is partially dehydrated when cooled below the freezing point of HW. Though this phenomenon is reversible, hysteresis is observed when the purple membrane is rehydrated upon reheating.^7^

Kundu *et al.* investigated water dynamics between DMPC bilayers using femtosecond mid-IR pump–probe spectroscopy and observed “fast” water near the phosphate portion and “slow” water near the choline portion.^8^ The dynamics of fast water had no significant effect on the phase transition of DMPC, whereas slow water slowed down with the phase transition of DMPC from the gel to the liquid crystalline phase.^8^ These previous studies described the effect of interactions between the phospholipid headgroup and the water molecules on their dynamics. Hishida *et al.* conducted small-angle x-ray scattering (SAXS) and revealed that an average of 28 water molecules was hydrated per DMPC molecule upon mixing different water concentrations with DMPC.^9^ They also investigated differences in fast water (rotational) dynamics between 1-palmitoyl-2-oleoyl-*sn*-glycero-3-phosphocholine (POPC) and 1-palmitoyl-2-oleoyl-*sn*-glycero-3-phosphoethanolamine (POPE) by means of terahertz time-domain spectroscopy (THz-TDS).^10^ The only differences between these phospholipid molecules were the chemical structures of the headgroups, where –NH_3_^+^ was attached to the headgroup of POPE, and –N(CH_3_)_3_^+^ was attached to the headgroup of POPC. The dynamics of fast water in the multilamellar vesicles of POPE was faster than that in the multilamellar vesicles of POPC. These results indicate that rotational water dynamics depends on lipid headgroup structures. However, a comprehensive picture of HW dynamics, including translational motion and its relationship to the phospholipid headgroup structure, remains to be revealed.

QENS is a cutting-edge experimental technique for investigating both the dynamics of lipid molecules and HW.^11^ QENS gives the dynamic structure factor, *S*(*Q,ω*), of molecules within a timescale of 10^−12^–10^−9^  s and a length scale of 10^−11^–10^−8^  m. The hydrogen atom has a large incoherent scattering cross section in which neutrons are isotropically scattered without any correlation with the surrounding structure, that is, they reflect the dynamics of the hydrogen atoms themselves.^12^ The self-correlated dynamics of water molecules was identified using selective deuteration in complex systems including lipid–water mixtures^3,13–18^ and a protein–water mixture^19^ because the incoherent cross section of deuterium is approximately 40 times smaller than hydrogen. Thus, observing the self-correlated dynamics of water or phospholipid molecules is possible by investigating incoherent QENS with selective deuteration. Several groups have devoted efforts to verifying the dynamics of HW in the vicinity of lipid membranes. König *et al.* investigated a mixture of perdeuterated DPPC (d_75_DPPC) and H_2_O or D_2_O deposited on a substrate.^14^ They showed that only rotational water molecules exist at low hydration levels and identified a translational motion similar to that of bulk water in the high hydration state. However, they found no motional anisotropy parallel or vertical to the lipid membrane. Swenson *et al.* prepared protiated DMPC-D_2_O, tail-deuterated DMPC (d_54_DMPC)-D_2_O, and d_54_DMPC-H_2_O and obtained HW dynamics upon subtracting the QENS data for d_54_DMPC-D_2_O from those of d_54_DMPC-H_2_O.^17^ They characterized the hydration dynamics as jump diffusion, where the diffusion constant was lower than that of bulk water by only a factor of two. Toppozini *et al.* analyzed QENS signals from HW in the vicinity of DMPC and confirmed the occurrence of anomalous and anisotropic nanoscale diffusion of HW.^16^

**FIG. 1.**
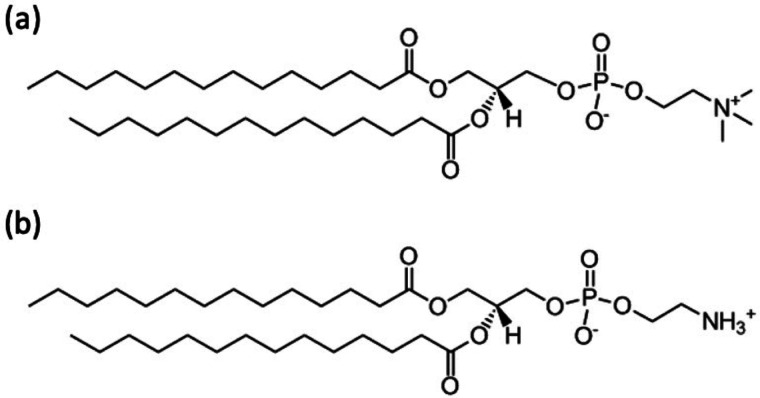
Chemical structure of (a) DMPC lipid and (b) DMPE lipid.

Recently, Yamada *et al.* reported the QENS results for HW incorporated between perdeuterated DMPC (d_67_DMPC) membranes in multilamellar vesicles.^13,20^ The QENS profiles for H_2_O could be characterized as the sum of three Lorentz functions, and these components were interpreted as (1) free HW whose diffusion coefficient (*D*) was comparable with that of bulk water, (2) loosely bound water, with a *D* one order of magnitude less than that of free HW, and (3) tightly bound water, with a *D* comparable with that of DMPC molecules.^13^ In addition, the effect of adding salts to the DMPC mixture on HW was investigated using QENS.^20^ The results showed that the number of tightly bound water molecules changed depending on the position of metal cation binding at the lipid headgroups, while the number of loosely bound water molecules remained unchanged. Figures 1(a) and 1(b) show the structure of DMPC and DMPE lipids, respectively.

The only difference between these phospholipids is the chemical structure of their headgroups. The headgroup of DMPC contains an N(CH_3_)_3_^+^, while the headgroup of DMPE contains –NH_3_^+^. Though the difference is small, an MD simulation study revealed that the –NH_3_^+^ in PE lipids is more strongly bonded to water than the N(CH_3_)_3_^+^ in PC lipids.^21^ The water molecules bonded to –NH_3_^+^ break hydrogen bonds with other water molecules. It means the hydrogen bond network among water molecules in DMPE may differ from that in DMPC. Thus, studying the HW dynamics near DMPE and comparing the results with that in DMPC are quite interesting.

In this study, we investigated the HW dynamics incorporated in DMPE bilayers and the headgroup dynamics of DMPE by means of QENS in order to clarify the relationship between the dynamical behavior of phospholipid headgroups and that of HW. The headgroup dynamics of DMPE was also studied using MD simulations. When combined, these results revealed that HW dynamics depends on the headgroup structure of lipid molecules.

## II. MATERIALS AND METHODS

### A. QENS and elastic intensity scan experiments

**FIG. 2.**
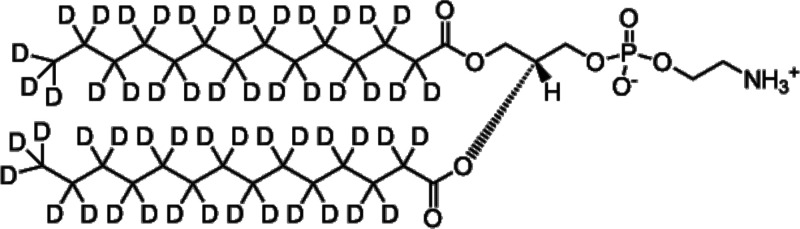
Chemical structure of d_54_DMPE.

Tail-deuterated DMPE (d_54_DMPE) was purchased from Avanti Polar Lipids, Inc. (Birmingham, AL, USA) and used without further purification. Figure 2 shows the chemical structure of d_54_DMPE.

Appropriate amounts of d_54_DMPE and D_2_O (99.9%, Merck & Co., Inc., Rahway, NJ, USA) or H_2_O were mixed in vials. The samples were wrapped in 43 × 35 mm aluminum foil, placed inside aluminum cells with an inner diameter of 14.0 mm and a thickness of 0.25 mm, and sealed in the cells with metal O-rings. These operations were conducted under a helium atmosphere with a relative humidity (RH) of 100% D_2_O or H_2_O conditions in a glove bag. The compositions of the QENS samples calculated from the preparation weight were 10 water molecules per DMPE molecule (d_54_DMPE-10D_2_O and d_54_DMPE-10H_2_O). Sample homogeneity was confirmed using SAXS experiments (see Fig. S1).^22,23^ The mass of each sample was approximately 30 mg.

The dynamical structure factor S(Q,ω) is the sum of two components: incoherent scattering originates from the self-correlation and dynamics of individual atoms, and coherent scattering carries information on the structure of a material and the collective dynamics of atoms. Since the incoherent scattering cross section of hydrogen atoms is quite large, the main contributions to QENS signals from a mixture of water and chain-deuterated lipids are from the self-correlation of water molecules and lipid headgroups. In the present mixture, the contributions of the headgroups of d_54_DMPE containing 12 hydrogen atoms and 10 HW molecules (20 hydrogen atoms) are comparable. Therefore, the QENS signals from the d_54_DMPE-10H_2_O should be interpreted in terms of both the lipid headgroup dynamics and the hydrated water dynamics, while those from the d_54_DMPE-10D_2_O reflect lipid headgroup dynamics. In this context, data on d_54_DMPE-10D_2_O were analyzed first to estimate the headgroup dynamics, and data on d_54_DMPE-10H_2_O were analyzed next using the fit parameters obtained in the first data analysis to extract the dynamics of the hydrated water molecules.

QENS experiments were carried out using a time-of-flight near-backscattering spectrometer DNA (BL-02) at the Materials and Life Science Experimental Facility (MLF) at the Japan Proton Accelerator Research Complex (J-PARC) in Tokai, Japan.^24,25^ The injected proton beam power incident on the neutron target was ∼600 kW. High-resolution and high-intensity (low-resolution) modes, with energy resolutions of 3.6 and 13 *μ*eV with or without an operating pulse shaping chopper (225 Hz in operation), respectively, were used to cover a wide energy transfer range. The covered Q-range was 0.17–1.82 Å^−1^. The QENS measurements of d_54_DMPE-10H_2_O and d_54_DMPE-10D_2_O were carried out at 368, 358, 343, 330, 313, and 300 K for both the high-resolution (energy resolution 3.6 *μ*eV) and high-intensity (energy resolution 13 *μ*eV) modes. The energy transfer ranges (Δ*E*) were −30 ≤ Δ*E* ≤ 60 *μ*eV for the high-resolution mode and −0.5 ≤ Δ*E* ≤ 1.5 meV for the high-intensity mode. The QENS signals from the samples measured at 85 K were used as resolution functions. The exposure times for the high-resolution and high-intensity modes were 3.5 and 1.5 h, respectively. The elastic intensities were also recorded during the temperature change in the QENS measurements using the high-resolution mode, and the heating and cooling rates were approximately 1 K min^−1^.

### B. MD simulations

**FIG. 3.**
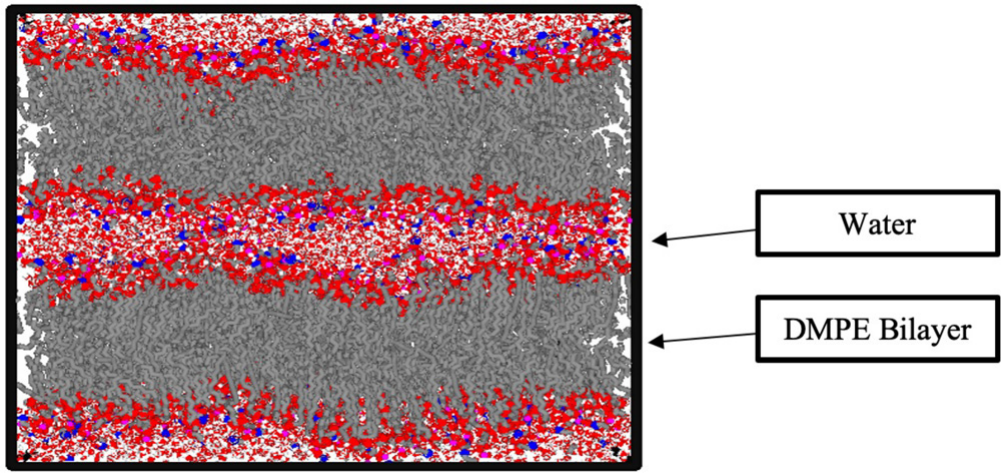
A typical snapshot from the MD simulation of DMPE-10H_2_O. Hydrogen atoms in the tails are depicted as transparent. The red, white, blue, purple, and gray spheres represent oxygen, hydrogen, nitrogen, phosphate, and carbon, respectively.

All-atomic MD simulations were performed on DMPE-10H_2_O at *T* = 300, 315, 330, 345, 360, and 375 K to reveal the dynamics of the DMPE headgroups. MD simulations were performed using the GROMACS^26,27^ version 2020.6 simulation package on the NPT ensemble (T is a constant temperature, P is a constant pressure, and N is a constant number of atoms). Pressure and temperature were regulated using the methods of Parrinello-Rahman^28^ and Nose^29^-Hoover,^30^ respectively. Phospholipid bilayer system configurations were assembled using Membrane Builder^31–33^ from Charmm-GUI.^34^ Two bilayers, consisting of a total of 1024 lipid molecules and 10 240 water molecules, were prepared, as shown in Fig. 3.

Newton's equation of motion was integrated using the leap-frog method with d*t* = 2 fs. The LINCS algorithm was executed on the hydrogen bond to increase d*t*.^35^ The SHAKE and RATTLE algorithms were used to calculate water dynamics.^36^ The smooth-particle mesh Ewald method was used to calculate long-range electrostatic interactions.^37^ The simulation reached a stable state after monitoring the total potential energy change for 130 ns. Data from 130 ns (after relaxation) to 200 ns were used for analysis.

## III. RESULTS AND DISCUSSION

### A. Elastic intensity scan

**FIG. 4.**
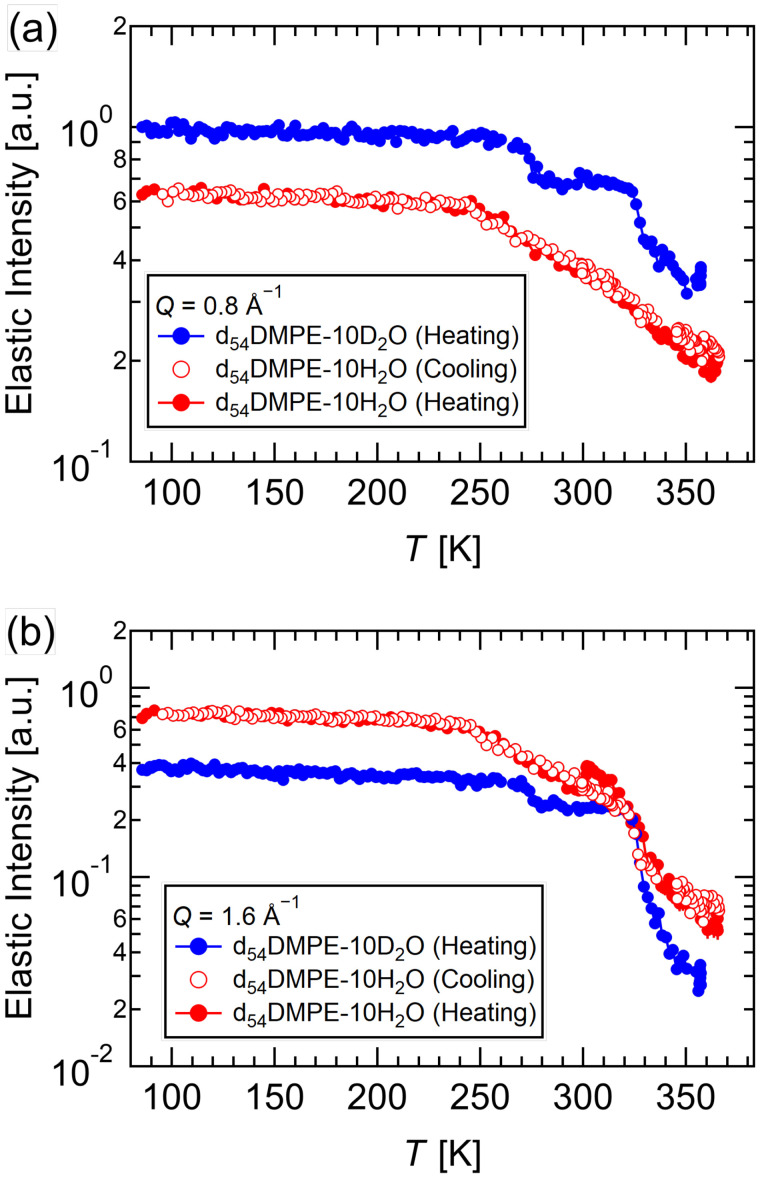
Elastic intensities of d_54_DMPE-10D_2_O and d_54_DMPE-10H_2_O at (a) *Q* = 0.85 and (b) *Q* = 1.6 Å^−1^. The blue-filled circles were obtained upon heating of d_54_DMPE-10D_2_O. The red open and filled circles were obtained upon cooling and heating of d_54_DMPE-10H_2_O, respectively.

Figure 4 shows the elastic scattering intensities of d_54_DMPE-10D_2_O and d_54_DMPE-10H_2_O at *Q* = 0.8 and *Q* = 1.6 Å^−1^, which were summed between *Q* = 0.375 and 1.325 Å^−1^ and *Q* = 1.375 and 1.725 Å^−1^, respectively. While most of the neutron scattering intensity at *Q* = 0.8 Å^−1^ was incoherent scattering, the intensity at *Q* = 1.6 Å^−1^ contains incoherent and coherent scattering from the deuterated alkyl chain corresponding to inter-chain ordering. Since the alkyl chain melts above the main transition temperature, the elastic scattering intensity at *Q* = 1.6 Å^−1^ is suitable to check the main transition temperature. The elastic intensities of both samples drastically changed at the same temperature, T ≈ 325 K [see Fig. 4(b)], a signature of the main transition. It is known that the main transition temperature varies depending on the water content. Thus, this result confirmed that the water content was the same in the d_54_DMPE-10D_2_O and d_54_DMPE-10H_2_O samples, as expected.

As mentioned earlier, the elastic scattering intensity at *Q* = 0.8 Å^−1^ [Fig. 4(a)] is due to incoherent scattering from immobile hydrogen atoms. The intensity of the d_54_DMPE-10D_2_O, in which most of the incoherent scattering came from lipid headgroups, showed drastic decreases at 273 and 325 K and continuous decreases above 325 K. On the other hand, the intensity of the d_54_DMPE-10H_2_O, in which the incoherent scattering includes the contributions from lipid headgroups and water molecules, showed a monotonic decrease above 250 K without a further jump at 325 K. When a harmonic oscillator is used to represent the thermal motion of molecules, the elastic intensity depends linearly on the temperature on a logarithmic scale. Any deviation from a straight line represents the possibility of relaxation and/or anharmonic oscillation in the sample. The change in the slope of the elastic intensity from d_54_DMPE-10H_2_O around 250 K is due to the melting of water because no change was observed in d_54_DMPE-10D_2_O. This result was consistent with the DSC results of Aoki and Kodama.^4^ They investigated the thermal properties of mixtures with different amounts of HW and observed ice melting peaks over a wide temperature range of around 250 K [Fig. 2(a), Ref. 4]. They suggested that the melting peaks observed for freezable water correspond to HW bound to lipid headgroups.^4^ The observed jump in the d_54_DMPE-10D_2_O at 273 K indicated that a kind of motion of these headgroups was unlocked upon the melting of water. Similarly, the melting of the alkyl chains also induced further motion of the headgroups. Because lipid headgroups are sandwiched between the HW and the alkyl chain, the interpretation that the lipid headgroup dynamics is affected by both the water and the alkyl chain is reasonable. On the contrary, water dynamics is insensitive to the alkyl chain dynamics. As described earlier, the elastic intensity from d_54_DMPE-10H_2_O contains information on the headgroup dynamics; however, the jump at 325 K may be suppressed by the superposition of the signal from water dynamics.

### B. Headgroup dynamics of DMPE

#### 1. QENS data analysis

**FIG. 5.**
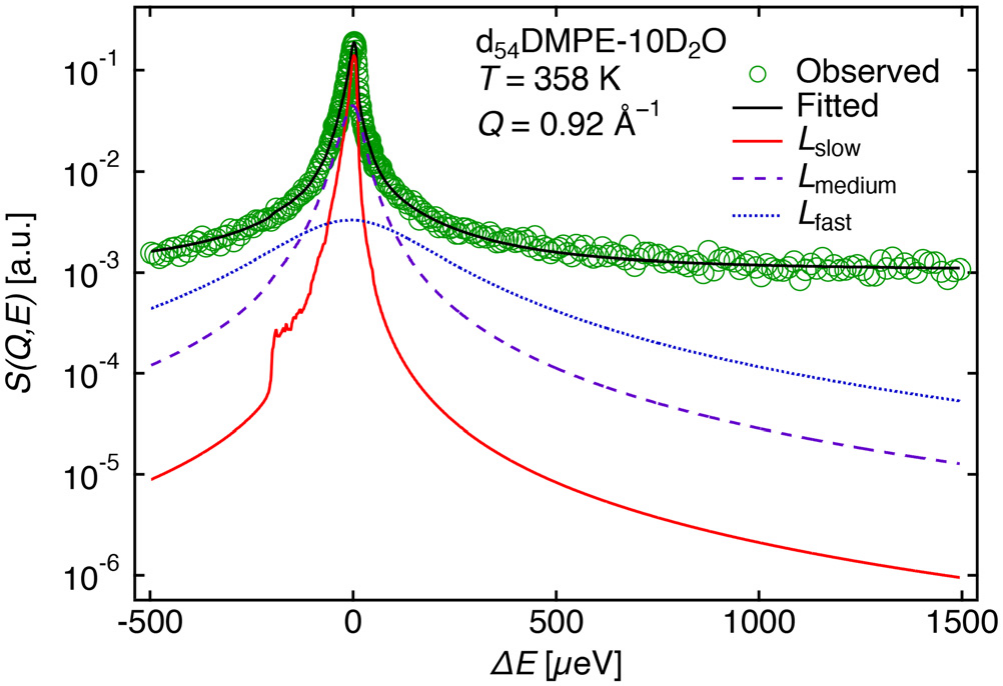
QENS profile of d_54_DMPE-10D_2_O at *Q* = 0.92 Å^−1^ at 358 K obtained using the high-intensity mode (13 *μ*eV energy resolution). The circles represent the experimental data, and the black line represents the experimental data fitted using Eq. (1a). The solid (red), dashed (violet), and dotted (blue) lines represent the $L_{\text{slow}}$, $L_{\text{medium}}$, and $L_{\text{fast}}$ of Eq. (1a), respectively.

From the QENS signal of d_54_DMPE-10D_2_O, the dynamic behavior of DMPE headgroups could be identified because the contribution of incoherent scattering from the headgroup of DMPE comprised approximately 86% of the total incoherent scattering. Figure 5 shows the QENS profile of d_54_DMPE-10D_2_O at *Q* = 0.92 Å^−1^ and *T* = 358 K (liquid crystalline phase) observed for the high-intensity mode (energy resolution Δ*E* = 13 *μ*eV, which corresponds to a time window of ∼97 ps), as a typical example. The QENS profile at 300 K (gel phase) is shown in the supplementary material (Fig. S2).^46^ Data could not be fit using the 1 or 2 Lorentz functions. The QENS profiles were well-fitted using the sum of three Lorentz functions, according to Eq. (1a),

$$\begin{array}{c} {S\left(Q,E\right)}_{\mathrm{DMPE}\;\text{head}}={\{A}_{\text{slow}}L_{\text{slow}}\left(\Gamma_{\text{slow}},\;E\right)+A_{\text{medium}}L_{\text{medium}}\left(\Gamma_{\text{medium}},\;E\right)\\ +A_{\text{fast}}L_{\text{fast}\;}(\Gamma_{\text{fast}},\;E)\}⊗R\left(Q,E\right)+BG; \end{array}$$ (1a)

$$L\left(\Gamma_{\alpha },E\right)=\frac{1}{\pi }\frac{\Gamma_{\alpha }}{E^{2}+{\Gamma_{\alpha }}^{2}},$$ (1b)

where *L*, *A*, *Γ*, *R*, *BG*, and ⊗ indicate the Lorentz function, amplitude (the coefficient of the Lorentz function), the half-width at half-maximum of the Lorentz function, the resolution function, the constant background, and the convoluted operator, respectively. Subscripts “slow,” “medium,” and “fast” are used to interpret the slow, medium, and fast modes of the DMPE headgroup, respectively. The equation of $S\left(Q,E\right)$ followed a textbook written by Bée^38^ and a journal article authored by Wanderlingh *et al.*^39^

**FIG. 6.**
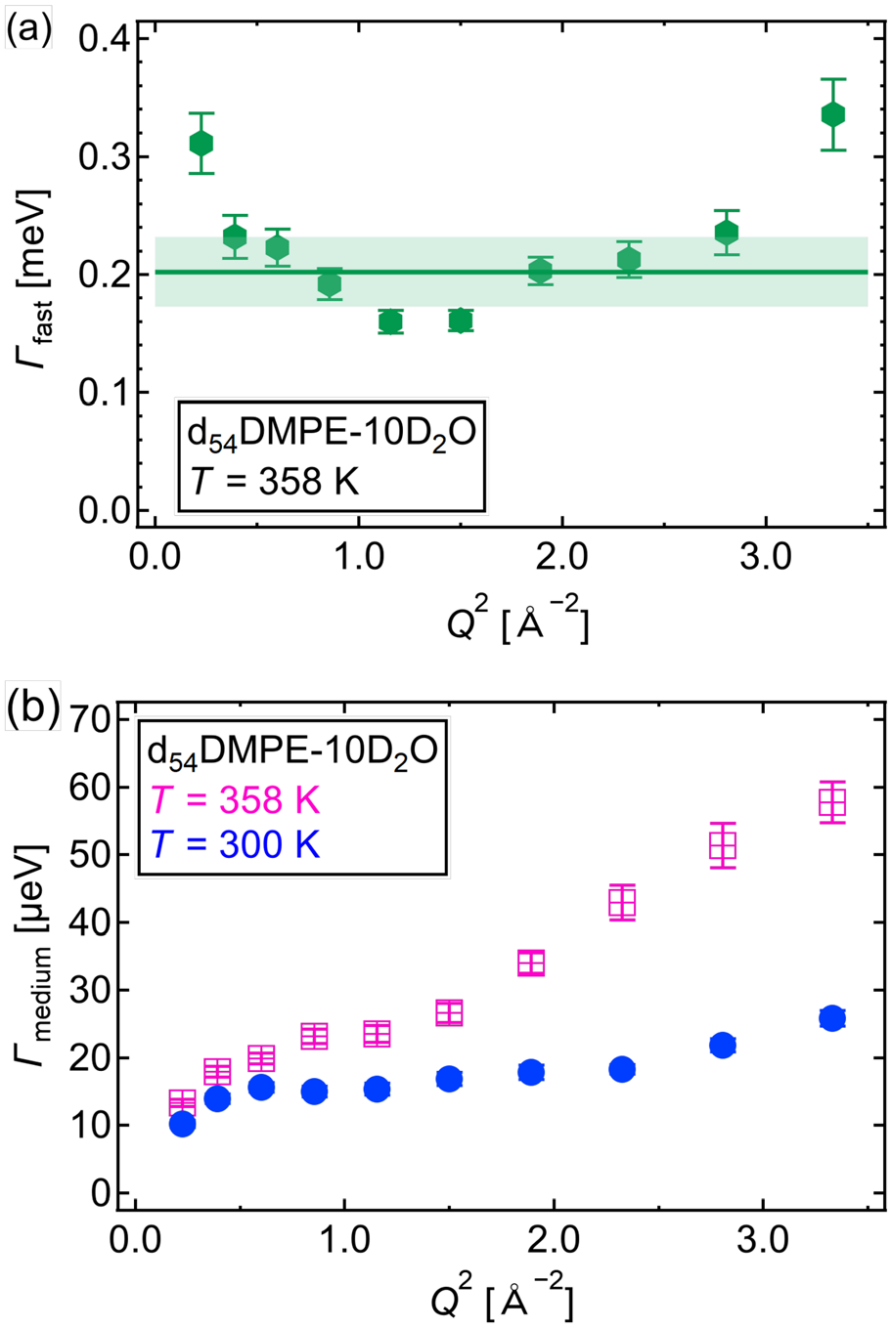
(a) Half-width at half-maximum (HWHM) values of the fast mode (*Γ*_fast_) vs *Q*^2^ of d_54_DMPE-10D_2_O, obtained using profile fitting to Eq. (1a) at 358 K. The solid line is the average of *Γ*_fast_ between *Q*^2^ = 0.39 and 2.80 Å^−2^. The shadow is the standard deviation. (b) HWHM values of the medium-speed mode (*Γ*_medium_) vs *Q*^2^ of d_54_DMPE-10D_2_O, obtained using profile fitting to Eq. (1a) at 358 K (pink-crossed squares) and 300 K (blue-filled circles).

Figure 6(a) shows *Γ*_fast_ vs *Q*^2^ of the headgroup of DMPE at 358 K (liquid crystalline phase). The *Γ*_fast_ values at *Q*^2^ = 0.22 and 3.33 Å^−1^ show larger error bars than others; thus, the discussion is continued by neglecting these two values. Except for the two *Γ*_fast_ values, *Γ*_fast_ appears to be independent of *Q*, which indicates that the fast mode of the DMPE headgroup could be considered as a local motion. The origin of the fast mode may relate to the rotational motion of the amine and/or methylene groups; however, this interpretation is an oversimplification considering the complicated chemical structure of the DMPE headgroup. The large deviation in the Γ values may be due to the complex motion of the headgroups; however, data were limited, and the quantity was insufficient for an interpretation.

Figure 6(b) represents *Γ*_medium_ vs *Q*^2^ at 358 K (liquid crystalline phase) and 300 K (gel phase). In the liquid crystalline phase and the gel phase, *Γ*_medium_ increased with increasing *Q*^2^, suggesting translational motion.

**FIG. 7.**
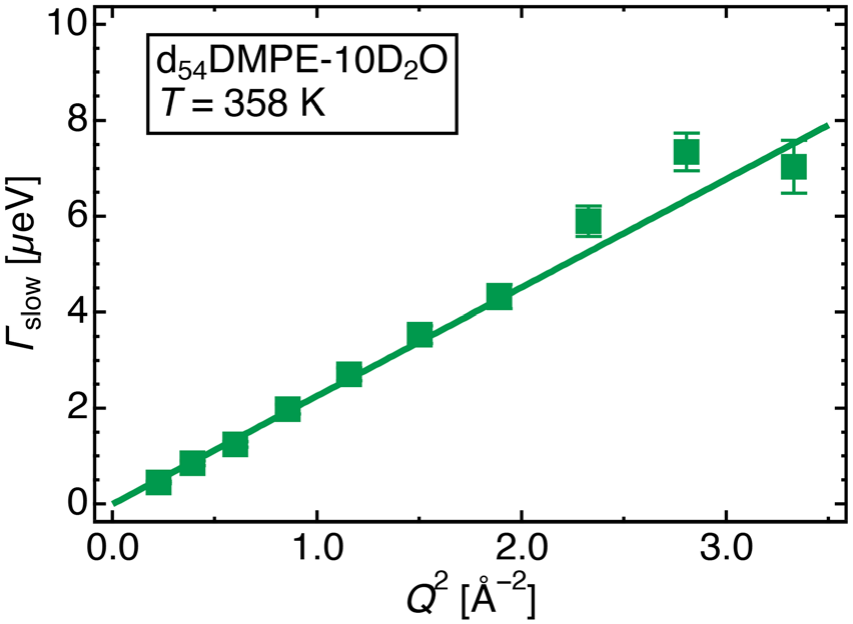
HWHM values of the slow mode (*Γ*_slow_) vs *Q*^2^ of d_54_DMPE-10D_2_O obtained by profile fitting to Eq. (2) at 358 K. The solid line is the fitted line described by Eq. (3) below and *D*_slow_ = (3.43 ± 0.05) × 10^9 ^ Å^2^ s^−1^.

In order to identify the slow mode of the DMPE headgroup, consistent data analyses were performed for both QENS profiles at both the high-intensity and high-resolution modes of Δ*E* = 3.6 *μ*eV, corresponding to a 350 ps timescale. The QENS profile obtained using the high-energy resolution mode is shown in Fig. S3 in the supplementary material.^46^ This profile can be fitted using the sum of two Lorentz functions as follows:

$${S\left(Q,E\right)}_{\mathrm{DMPE}\;\text{head}\;\mathrm{HR}}={\{A}_{\text{slow}}L_{\text{slow}}\left(\Gamma_{\text{slow}},\;E\right)+A_{\text{medium}}L_{\text{medium}}(\Gamma_{\text{medium}},\;E)\}\;⊗\;(R(Q,E)+BG,$$ (2)

where the parameters of the medium-speed mode are fixed using the fitting results of the high-intensity mode. The *Q*^2^ dependence of the obtained *Γ*_slow_ is shown in Fig. 7.

The *Γ*_slow_ vs *Q*^2^ relationship for d_54_DMPE-10D_2_O in Fig. 7 follows Fick's law, as follows:

$$\Gamma_{\text{slow}}=D_{\text{slow}}\;Q^{2},$$ (3)

where *D*_slow_ is the *D* of the DMPE headgroup slow mode.

The *Q*^2^ dependence of *Γ*_slow_ vs d_54_DMPE-10D_2_O for other temperatures is shown in the supplementary material (Fig. S4).^46^

**FIG. 8.**
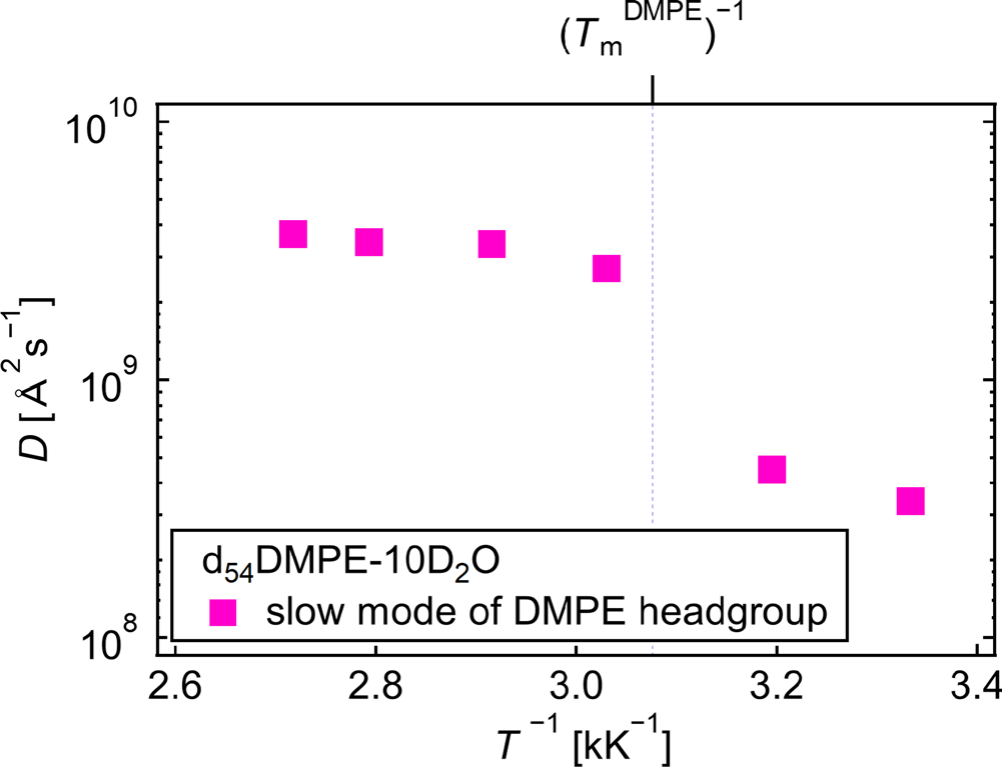
Arrhenius plot of diffusion coefficients of the DMPE headgroup slow mode.

Figure 8 shows the Arrhenius plot of the *D*s of the slow mode in d_54_DMPE-10D_2_O (the obtained values are summarized in Table S1). ( $T_{m}^{\mathrm{DMPE}}$)^−1^ represents the inverse of the main phase transition temperature (*T* = 325 K) estimated from the elastic intensity scan. The *D*s in the liquid crystalline phase were comparable with previous results for protiated DMPC-35D_2_O, *D* = 3.8 × 10^9^ Å^2^s^−1^ at *T* = 316 K (liquid crystalline phase)^13^ as well as other values in the literature.^15,18,40–42^ The *D* decreased sharply at $T_{m}^{\mathrm{DMPE}}$, which indicates that the DMPE headgroup's slow mode is strongly related to the phase transition of DMPE. The diffusion of the slow mode of lipid molecules is slowed in the gel phase; this result is consistent with a previously published study of DMPC using QENS.^15^ They explained that the lipid molecules are well-ordered and densely packed in the gel phase, which slowed the lateral diffusion of lipid molecules. The previous study endorses that the slow mode of the DMPE headgroup relates to the cooperative motion of DMPE molecules within the membrane. Thus, it can be concluded that the DMPE membrane dominates the *D* of the slow mode of the DMPE headgroup.

#### 2. MD simulations

An all-atomic MD simulation was performed in order to investigate the origin of medium-speed and fast modes of the headgroup of DMPE. First, we performed an MD simulation to check the tail orders of DMPE in order to confirm its phase transition. In Fig. S5 (see the supplementary material, Ref. 46), the tail order was highest at 300 K. Upon an increase in temperature from 300 to 330 K, the order suddenly decreased. Subsequently, the rate of decrease in the tail order became small in the temperature range of 330–375 K. This indicates that the transition of DMPE from the gel phase to the liquid crystalline phase occurred in the 300–330 K temperature range. The result for the main phase transition temperature obtained using MD simulation was consistent with that of the elastic scan (see Fig. 4). This result confirms the validity of the MD simulations.

##### a. Medium-speed mode of the DMPE headgroup

**FIG. 9.**
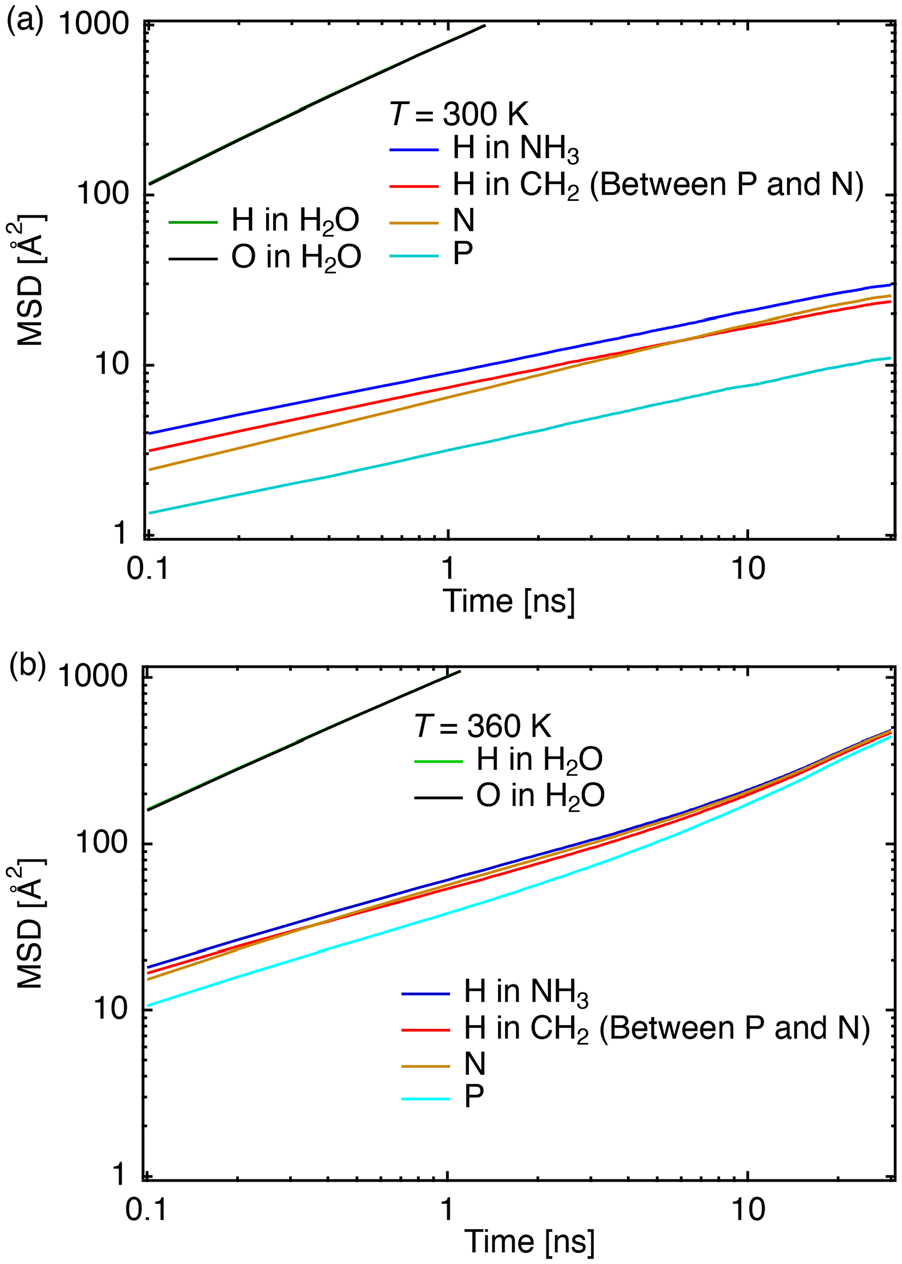
(a) Mean-square displacement (MSD) in Å^2^ vs time (ns) of atoms in the DMPE headgroup at 300 K. The lines of “H in H_2_O” and “O in H_2_O” overlap completely; (b) MSD vs time (ns) of atoms in the DMPE headgroup at 360 K. The lines of “H in H_2_O” and “O in H_2_O” overlap completely.

Figures 9(a) and 9(b) show the mean-square displacement (MSD) in Å^2^ vs time (ns) of atoms in the DMPE headgroup at 300 (gel phase) and 360 K (liquid crystalline phase), respectively.

**FIG. 10.**
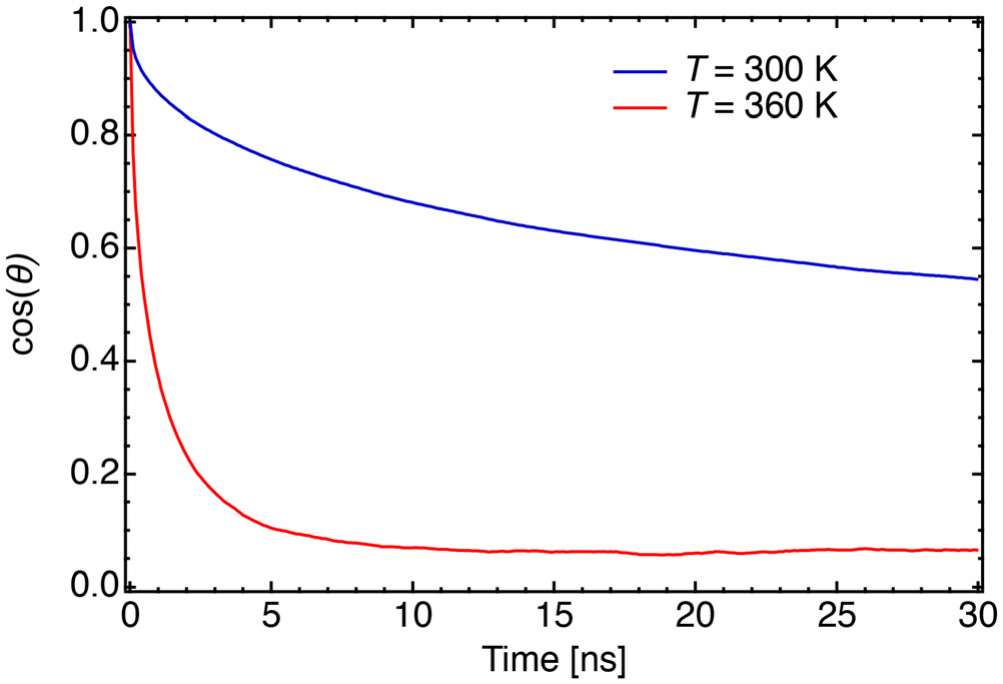
Rotational correlation ⟨cos *θ*(*t*)⟩ of the headgroup in DMPE at 300 K (blue line) and 360 K (red line).

At 300 K, the diffusion of water molecules is much faster than that of phospholipids, and the slopes of the MSDs in phospholipid atoms were approximately identical. This tendency indicates the diffusion of whole DMPE molecules has a nanosecond timescale. At 360 K (above $T_{m}^{\mathrm{DMPE}}$), as shown in Fig. 9(b), the tendency was identical to that at 300 K for 0.1–6.0 ns. The entire molecule's motion was mainly responsible for the diffusion. Remarkably, the slope increased after 6 ns, which was not observed at 300 K. To fully reveal the different behaviors, the rotational correlation of the headgroup was calculated. The rotational correlation was calculated from the time correlation function of the direction of the headgroup: cos *θ*(*t*), where *θ*(*t*) is the angle between the vector from P to N atoms in the headgroup at times *t* and *t* = 0 (initial time). Figure 10 shows that ⟨cos *θ*(*t*)⟩ at 300 K (gel phase) is approximately 0.55 at 30 ns, and the correlation is unrelaxed. This slow relaxation of the N atoms means that these atoms were fixed on a nanosecond timescale. It is natural that positively charged N atoms interact intermolecularly with negatively charged P atoms as a result of electrostatic interactions. The strong intermolecular interaction between P and N atoms was consistent with whole molecule motion, as shown in Fig. 9(a), where the MSD slopes of atoms in the lipid headgroup were approximately identical. At 360 K (liquid crystalline phase), ⟨cos *θ*(*t*)⟩ sharply decreased in 5 ns. Above $T_{m}^{\mathrm{DMPE}}$, the rotational correlation was relaxed on the order of several nanoseconds. Therefore, the change in the slope of the diffusion at the liquid crystalline phase was caused by headgroup rotation (detaching of P and N atoms). The diffusion time of the headgroup rotational motion at 360 K ranged from 6 to 70 ns, which the present QENS experiments could not establish.

In the nanosecond range, the similar MSD slopes suggest that the diffusion originates from fluctuations in the DMPE molecule due to the intermolecular connection arising from the electrostatic interactions between P and N atoms. Figure 6(b) shows *Γ*_medium_ increased with increasing *Q*^2^, indicating that the medium-speed mode of the headgroup of DMPE was translational. The QENS and MD simulation results helped to conclude that the medium-speed mode of the headgroup of DMPE fluctuates the entire DMPE molecule from its original position by means of thermal fluctuations.

##### b. Fast mode of the DMPE headgroup

**FIG. 11.**
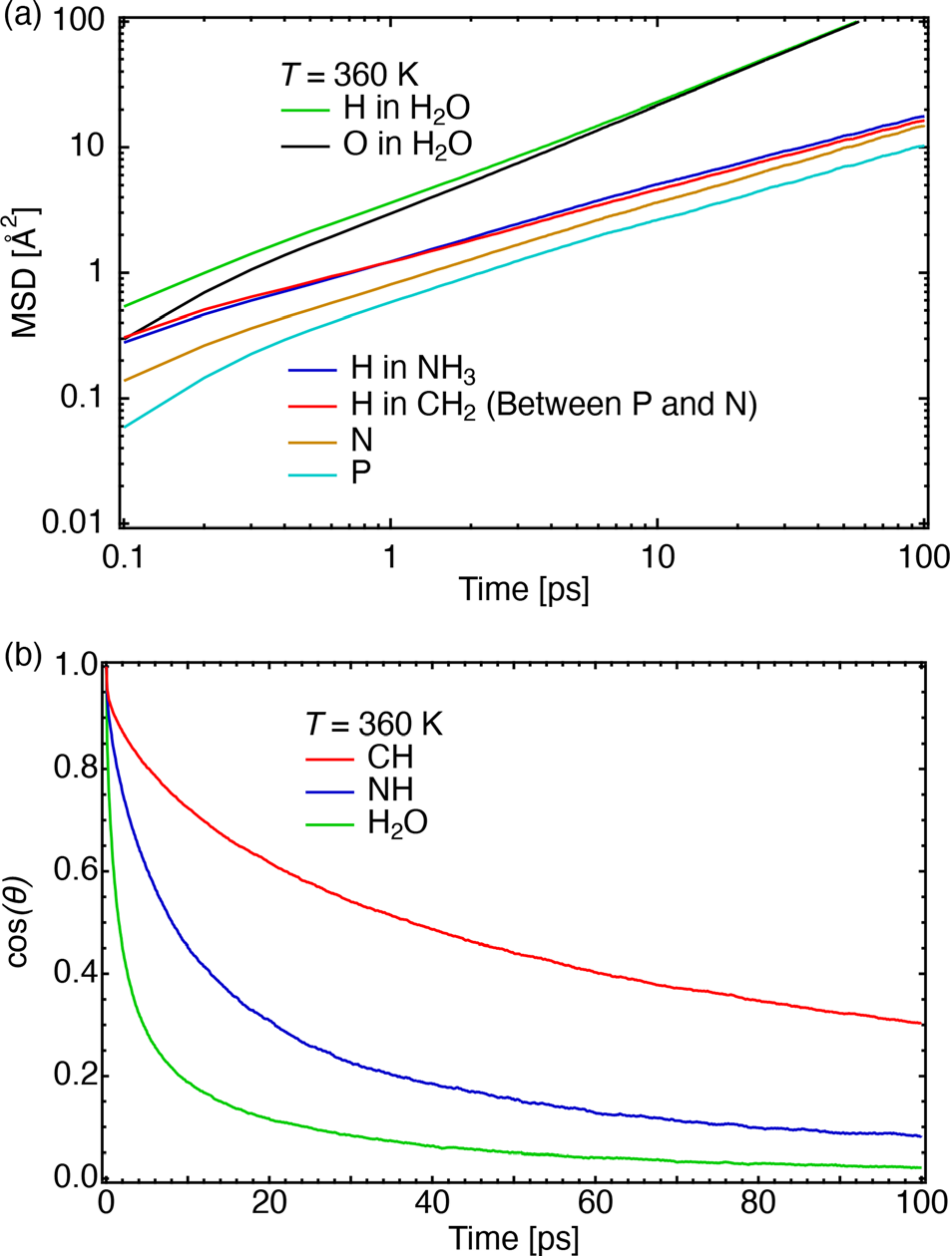
(a) MSD (Å^2^) vs time (ps) in the DMPE headgroup at 360 K; (b) rotational correlation, ⟨cos *θ*(*t*)⟩, of hydrogen atoms around C and N atoms in the headgroup and water molecules at 360 K.

Figure 11(a) represents the MSD of the DMPE headgroup as a function of time in ps at 360 K. The diffusion of water molecules is faster than that of phospholipid molecules. The MSDs of hydrogen atoms connected to C and N atoms in the headgroup are approximately identical. The MSD at 300 K, Fig. S6(a) in Ref. 46, shows that the diffusion of the hydrogen atoms bonded to –NH_3_^+^ groups was faster than those bonded to methylene (–CH_2_–) groups. To identify the mode, rotational correlations were calculated [Fig. 11(b)]: the vector from C to H atoms in the headgroup (–CH_2_–), the vector from N to H atoms in the headgroup (–NH_3_^+^), and the dipole vector in the water molecule (H_2_O) were used to decide the angles. The H atoms in –NH_3_^+^ rotated in the gel as well as the liquid crystalline phases [Fig. 11(b)]. Figure S6(b) in Ref. 46 shows an instance when the rotational correlation decreased by an order of 10 ps. A greater decrease in the rotational correlation of H atoms occurred in –CH_2_– at 300 K than at 360 K. These differences appear in Fig. 11(a) and the supplementary material [Fig. S6(a)].^46^ The MD simulation observed rotational motions on the order of 10 ps. This type of fast localized motion in the headgroup of DMPE observed using QENS at 358 K is shown in Fig. 6(a). Therefore, we suggest that the origin of the fast mode observed using QENS is the rotation of H atoms around N and C atoms in a DMPE headgroup.

Recently, Matsuo *et al.* investigated the motion of DMPC and tail-deuterated DMPC (d_54_DMPC) lipid by means of QENS and explained that intermediate motion corresponds to collective rotational diffusion, internal flip-flop motion, and internal rotation of the headgroup.^43^ The *Γ* of the intermediate motion of the d_54_DMPC by QENS [Ref. 43, Fig. 5(d)] for their case was between 0.2 and 0.8 meV. While in this present interpretation of the fast mode of the DMPE headgroup, the *Γ*_fast_ range is approximately between 0.1 and 0.4 meV, which is consistent with their intermediate motion. Thus, it can be suggested, based on this MD simulation study, that the fast mode of the DMPE headgroup could be the rotation of H atoms around N and C atoms in the DMPE headgroup.

### C. Water dynamics via QENS

**FIG. 12.**
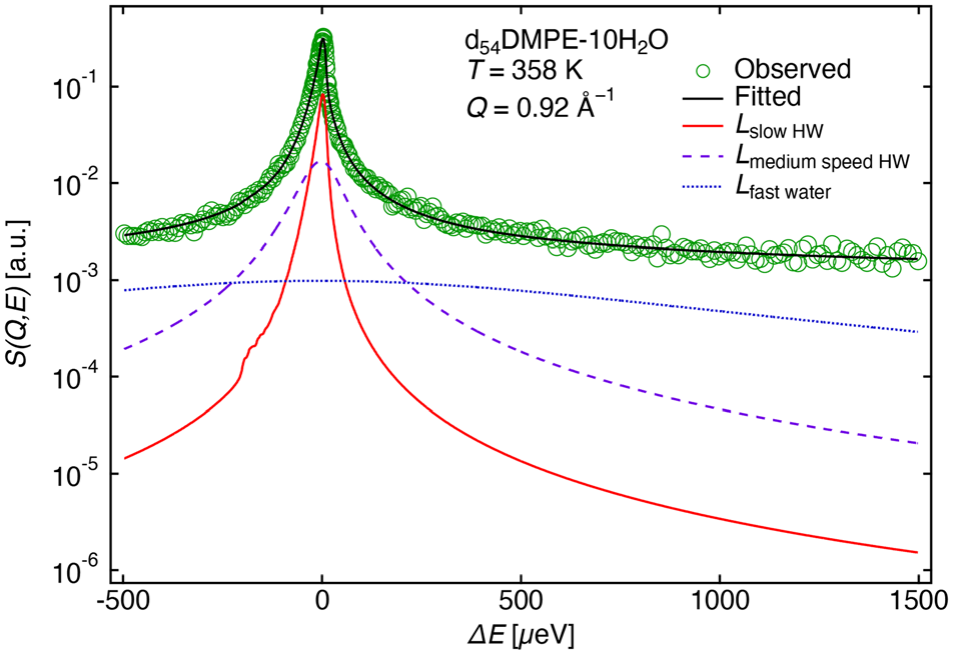
QENS profile of d_54_DMPE-10H_2_O at *Q* = 0.92 Å^−1^ and 358 K. The circles represent the experimental data, and the solid black line is the curve fit of the data using Eq. (4). The dotted, dashed, and solid red lines represent *L*_fast water_, *L*_medium speed HW_, and *L*_slow HW_ of Eq. (4), respectively.

Figure 12 shows the observed QENS profile and the fitted result for d_54_DMPE-10H_2_O for the high-intensity mode (Δ*E* = 13 *μ*eV) at 358 K. The observed QENS profiles were fitted using the sum of the contributions from DMPE headgroups and water as Eq. (4), following the methods in previously published studies,^13,17,20^

$$S\left(Q,E\right)=\left\{{S\left(Q,E\right)}_{\mathrm{DMPE}\;\text{head}}+{S\left(Q,E\right)}_{\text{water}}\right\}\;⊗\;R\left(Q,E\right)+BG;$$ (4a)

$${S\left(Q,E\right)}_{\mathrm{DMPE}\;\text{head}}=A_{\text{slow}}L_{\text{slow}}\left(\Gamma_{\text{slow}},E\right)+A_{\text{medium}}L_{\text{medium}}\left(\Gamma_{\text{medium}},E\right)+A_{\text{fast}}L_{\text{fast}}\left(\Gamma_{\text{fast}},E\right);$$ (4b)

$$\begin{array}{c} {S\left(Q,E\right)}_{\text{water}}=A_{\text{slow}\;\mathrm{HW}}L_{\text{slow}\;\mathrm{HW}}\left(\Gamma_{\text{slow}\;\mathrm{HW}},E\right)\\ {+A}_{\text{medium}\;\text{speed}\;\mathrm{HW}}L_{\text{medium}\;\text{speed}\;\mathrm{HW}}\left(\Gamma_{\text{medium}\;\text{speed}\;\mathrm{HW}},E\right)\\ {+A}_{\text{fast}\;\text{water}}L_{\text{fast}\;\text{water}}\left(\Gamma_{\text{fast}\;\text{water}},E\right). \end{array}$$ (4c)

During fitting, three distinct modes of water dynamics were categorized as (i) slow HW, (ii) medium-speed HW, and (iii) fast HW. All parameters in ${S\left(Q,E\right)}_{\mathrm{DMPE}\;\text{head}}$ were fixed using the fitting results of d_54_DMPE-10D_2_O. For fitting QENS profiles with different resolutions, the QENS profiles of the high-resolution mode (Δ*E* = 3.6 *μ*eV) were analyzed first to obtain the *Γ*_slow HW_. The QENS profile of the high-resolution mode is shown in the supplementary material (Fig. S7),^46^ where fast and medium-speed modes were neglected in the fitting because the energy window was too narrow to observe these modes. The QENS profiles for the high-intensity mode (Δ*E* = 13 μeV) were then analyzed by fixing the values of $\Gamma_{\text{slow}\;\mathrm{HW}}$ except for very few Q values (in low Q region) depending on the proper QENS profile fitting.

**FIG. 13.**
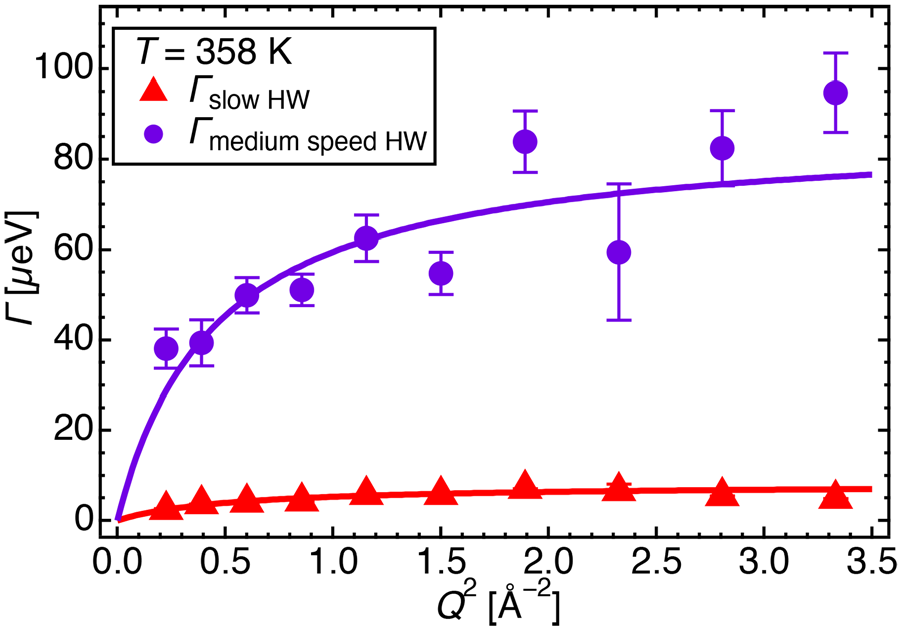
HWHM values of the slow and medium speed modes vs *Q*^2^ for d_54_DMPE-10H_2_O, obtained by profile fitting to Eq. (4a) at T = 358 K. The red triangles and blue circles represent *Γ*_slow HW_ and *Γ*_medium speed HW_ in Eq. (4a), respectively. The solid lines on *Γ*_slow HW_ and *Γ*_medium speed HW_ represent the fitted results obtained from Eq. (5).

Typical *Q*^2^ dependences of *Γ*_slow HW_ and *Γ*_medium speed HW_ at 358 K are shown in Fig. 13 (data for other temperatures are shown in Fig. S8 in the supplementary material).^46^ These dependencies increased with *Q*^2^, indicating that both slow and medium-speed modes were translational. These modes followed the jump-diffusion model,^13,20^ as shown by the solid lines in Fig. 13.

Equation (5) gives the expression of the jump-diffusion model for *Γ*

$$\Gamma =\frac{{DQ}^{2}}{1+{DQ}^{2}\tau_{0}},$$ (5)

where *D* and $\tau_{0}$ are the diffusion coefficient and mean residence time, respectively. The obtained *D* and $\tau_{0}$ values at the measured temperatures are listed in Table S2 of the supplementary material.^46^

**FIG. 14.**
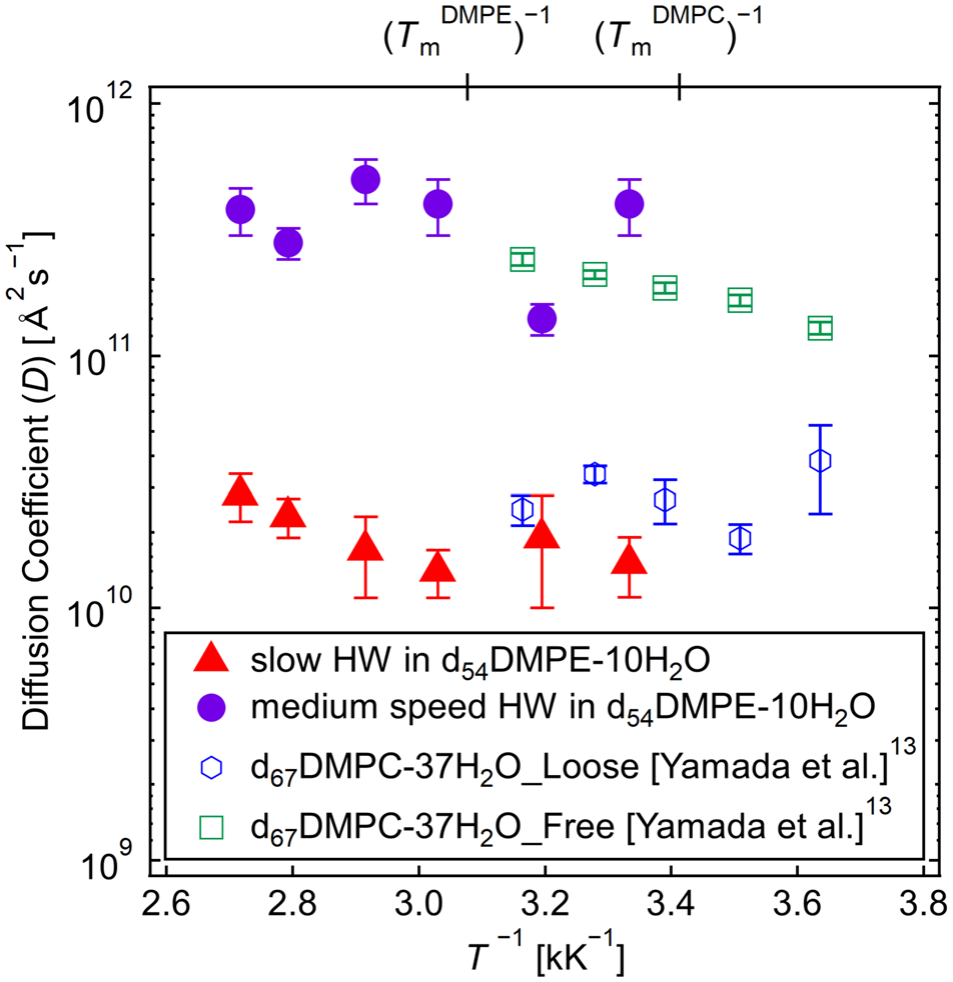
Arrhenius plots of the diffusion coefficients of HW in d_54_DMPE-10H_2_O obtained from Eq. (5). The filled red triangles and violet circles represent the diffusion coefficients of the slow- and medium-speed modes of HW between the DMPE membranes, respectively. The open squares and hexagons are the diffusion coefficients of the free and loosely bound HW between the DMPC membranes, respectively.^13^ ( $T_{m}^{\mathrm{DMPE}}$)^−1^ and ( $T_{m}^{\mathrm{DMPC}}$)^−1^ represent the inverse values of the phase transition temperatures for d_54_DMPE-10H_2_O and d_67_DMPC-37H_2_O,^13^ respectively.

Figure 14 shows Arrhenius plots of slow- and medium-speed HW *D*s between DMPE bilayers at the measured temperatures. In addition, the previous results for the *D*s of loosely bound and free HW between DMPC bilayers were plotted (Fig. 9 in Ref. 13) to compare them with the present results.

The *D*s of slow HW between DMPE bilayers are similar to that of loosely bound HW between DMPC bilayers.^13^ At 313 K, medium-speed HW shows similar dynamics compared with free HW in DMPC.^13^ It may be an artifact because the five other measured temperatures show that the *D*s of medium-speed HW in DMPE are faster than those in DMPC. Thus, it may be concluded concerning the five temperatures that medium-speed HW in DMPE is faster than free HW in DMPC.^13^

In the present study of d_54_DMPE-10H_2_O, tightly bound HW could not be determined. There were two possible reasons for this. First, the volume of water in the experimental sample was too small, and insufficient QENS signals from tightly bound water were included. Second, the tightly bound water dynamics could have been of the same order of magnitude as the slow mode of the DMPE headgroup; hence, separating them from the QENS signals was impossible.

**FIG. 15.**
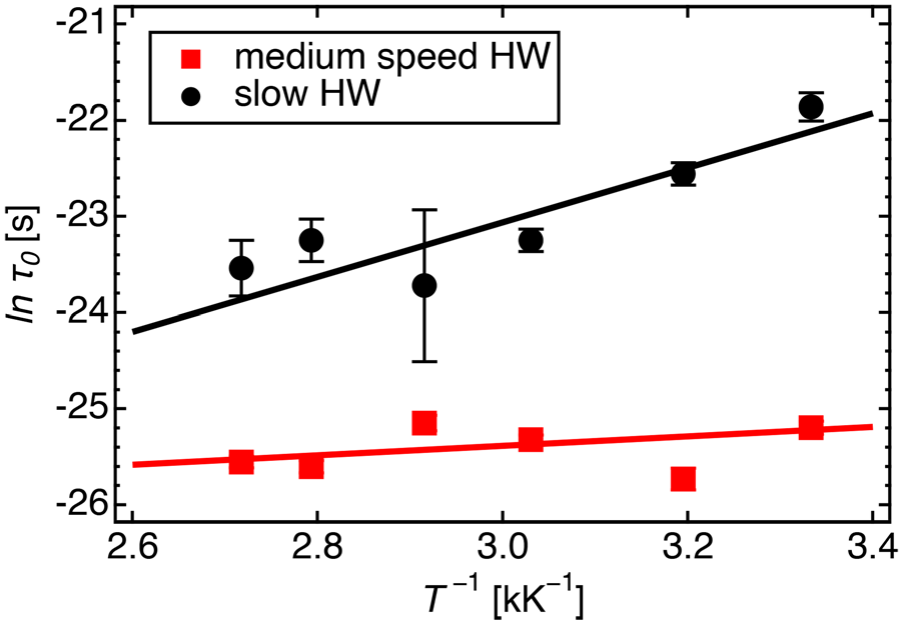
Arrhenius plots of the mean residence times of the diffusive translational modes of slow- and medium-speed HW in d_54_DMPE-10H_2_O, obtained from Eq. (5) for the six different measured temperatures.

The activation energies of slow- and medium-speed modes of HW between DMPE bilayers were compared with loosely bound and free HW between DMPC bilayers, respectively. The activation energy for mean residence time, $E_{a}$( $\tau_{0}$), was calculated from Arrhenius plots of the $\tau_{0}$ of medium-speed and slow HW modes of d_54_DMPE-10H_2_O (Fig. 15). The slope of the fitted line equals − $E_{a}$( $\tau_{0}$)/*R*, where *R* is the universal gas constant.

The activation energies, $E_{a}$( $\tau_{0}$), of medium-speed HW and slow HW between DMPE bilayers were 4 ± 1 and 23 ± 3 kJ mol^−1^, respectively. The $E_{a}$( $\tau_{0}$) values of free and loosely bound water between DMPC bilayers were 19.0 ± 2.5 and 27.5 ± 3.2 kJ mol^−1^, respectively.^13^ The activation energy of slow HW between DMPE bilayers was similar to that of loosely bound water between DMPC bilayers. In contrast, the activation energy of medium-speed HW between DMPE bilayers was significantly lower than that between DMPC bilayers. In our previous study, water dynamics between 1,2-dioleoyl-sn-glycero-3-phosphocholine (DOPC, a typical PC lipid) and 1,2-dioleoyl-sn-glycero-3-phosphoethanolamine (DOPE, a typical PE lipid) bilayers was investigated using MD simulations.^21^ The MD simulation results showed different hydrogen-bonded networks between the PC and PE lipids. The number of hydrogen bonds among water molecules around the PE lipid was less than that around the PC lipid. That MD simulation revealed that strong hydration between –NH_3_^+^ groups and water molecules breaks the hydrogen-bonded networks of water molecules in the case of the PE lipid. The results of QENS and our previous MD simulation study^21^ suggest the possibility that the hydrogen bonds among medium-speed HW molecules between DMPE bilayers are more distorted. Therefore, it can be concluded that the molecular arrangement of medium-speed HW molecules in DMPE could differ from that in DMPC.

**FIG. 16.**
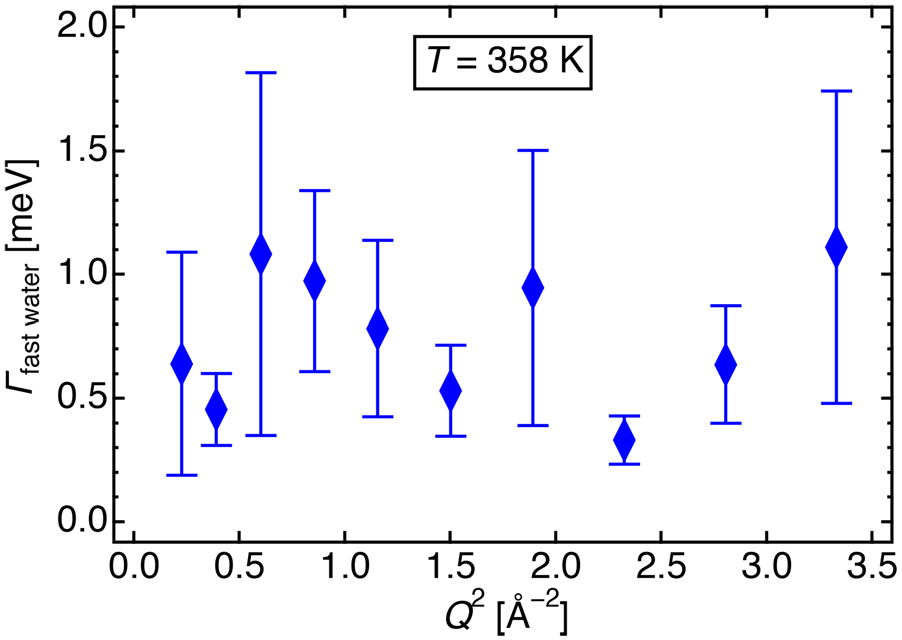
*Γ*_fast water_ vs *Q*^2^ for d_54_DMPE-10H_2_O, obtained by profile fitting to Eq. (4a) at 358 K.

Figure 16 shows the *Q*-dependence of HWHM of the fast mode of water, obtained from fitting these data to Eq. (4a) at *T* = 358 K.

Since *Γ*_fast water_ is independent of *Q*, the fast mode represents a localized motion. König *et al.* indicated, using QENS and NMR, that fast water molecules showed only rotational motion in the case of a low hydration level at four water molecules per DPPC molecule.^14^ Swenson *et al.* investigated water dynamics in the vicinity of DMPC bilayers using QENS.^17^ Their findings showed that the observed water dynamics was primarily rotational, especially at low temperatures and hydration levels. In this study, the number of water molecules was 10 per lipid molecule, and this number can be reasonably interpreted the fast mode as a rotational motion.

**FIG. 17.**
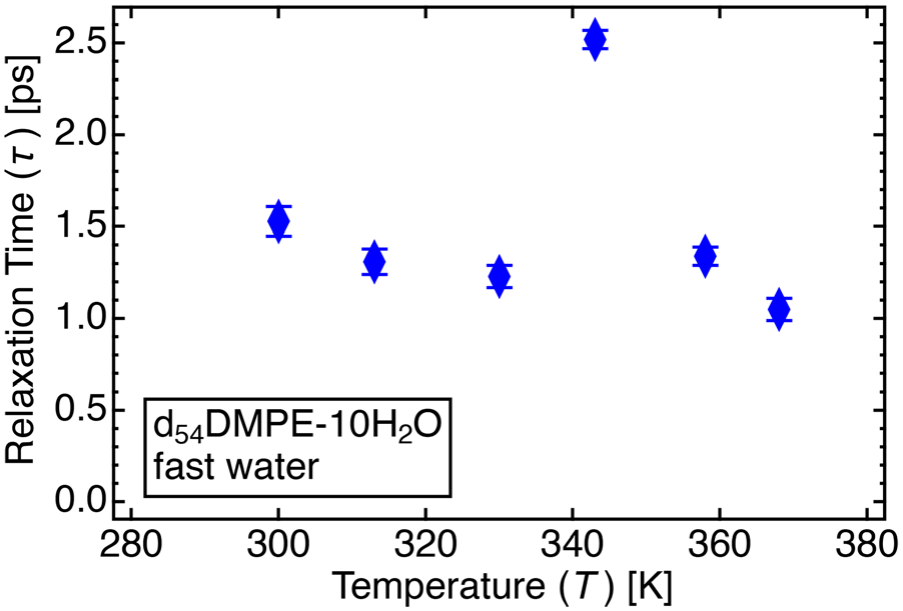
Temperature dependence of the relaxation time ( τ) of fast water in d_54_DMPE-10H_2_O. The relaxation time was calculated using Eq. (6).

From the average value of *Γ*_fast water_ (average energy dissipation, *E*), the relaxation time (*τ*) can be calculated as

$$\begin{array}{c} E=\hbar \omega =\hbar \frac{1}{\tau };\\ \tau =\frac{\hbar \;}{E}, \end{array}$$ (6)

where ω is the angular frequency. *Γ* vs *Q*^2^ plots of fast water in d_54_DMPE-10H_2_O, obtained upon profile fitting using Eq. (4a) of data measured at other temperatures, are shown in the supplementary material (Fig. S9).^46^ Those results also indicate that *Γ*_fast water_ is independent of *Q*. The relaxation time of the rotational motions was calculated from the average values of *Γ*_fast water_. The temperature dependence of the relaxation times on the rotational motion is shown in Table S3 in the supplementary material^46^ and depicted in Fig. 17. The relaxation time was calculated using Eq. (6).

The relaxation time is less than or approximately equal to 2.5 ps within the 300–368 K temperature range. The reason for the high value at *T* = 343 K is unknown, and it could have been due to an artifact; therefore, these data points were neglected in the discussion later. Although the relaxation time decreased with increasing temperature, the activation energy was very low. The phase transition from the gel phase to the liquid crystalline phase had no significant effect on the rotational dynamics of the water molecules, which was consistent with the observed rotational water dynamics in DPPC.^14^ The observed relaxation time of rotational water between DMPE bilayers was approximately six times faster than in DMPC^44^ and more than three times faster than in POPC,^10^ investigated using THz-TDS. Thus, water molecules behave differently at the phospholipid membrane surfaces depending on the charge of the phospholipids. This result was consistent with the MD simulation study, where the rotational water dynamics between DOPE (PE lipid) bilayers was faster than those between DOPC (PC lipid) bilayers.^21^

In our previous experiment on d_67_DMPC-37H_2_O,^13^ it was assumed that the dynamics of the DMPC molecule was related to the HW dynamics between DMPC bilayers. However, the headgroup dynamics of DMPC could not be separated because fully protiated DMPC was used, and measurements were made at only one temperature, namely, 306 K. Herein, the headgroup dynamics of DMPE from d_54_DMPE-10D_2_O was successfully characterized using QENS in the temperature range 300–368 K. We also clarified HW dynamics, where the *Γ* values of water were similar to those of the headgroup. Since water molecules and the phospholipid headgroup interact with each other, there is a possibility that the water and headgroup dynamics are influenced by each other. Wood *et al.* investigated the coupling dynamics of the purple membrane, HW, and protein dynamics using elastic incoherent neutron scattering, specific deuteration, and MD simulations. They concluded that HW dynamics is not directly coupled to membrane motions on the same timescale at temperatures <260 K.^45^ However, experimental studies should be conducted on a large temperature range to establish it. It can be concluded from the present study and previous studies^13,44^ that water dynamics near the lipid headgroup depend on the charge of the lipid headgroup.

## IV. CONCLUSIONS

The phospholipid headgroup dynamics of DMPE and the HW dynamics between DMPE bilayers using QENS, with a wide energy transfer range that covered three orders of magnitude of timescale, were investigated. Slow, medium-speed, and fast modes of headgroup motion were observed using tail-deuterated lipids (d_54_DMPE-10D_2_O). Based on the results of all-atomic MD simulations, it was decided that the origins of the slow, medium-speed, and fast modes were fluctuations in the DMPE membrane, fluctuations in the entire DMPE molecule, and the rotation of hydrogen atoms around N and C atoms in the headgroup of DMPE, respectively. Using selectively deuterated samples (d_54_DMPE-10H_2_O), water dynamics between DMPE bilayers was categorized into slow, medium-speed, and fast modes. Our comparison of the results with previous QENS results of HW dynamics between DMPC bilayers is summarized as follows:

(1) Diffusion of slow HW between DMPE bilayers was similar to that of loosely bound HW between DMPC bilayers and an order of magnitude less than that in bulk water.
(2) The diffusion of medium-speed HW between DMPE bilayers was faster than that of free HW between DMPC bilayers. Their activation energies were also different, suggesting the presence of different hydrogen-bonded networks of HW molecules around the phospholipids.
(3) The relaxation time of rotational water between DMPE bilayers was approximately six times faster than that in DMPC.

We revealed that fast (rotational) water dynamics depends on phospholipid headgroup structures. The *D* of slow HW dynamics in DMPE is similar to loosely bound HW dynamics in DMPC, while the *D*s of medium-speed HW dynamics are faster compared with free HW dynamics in DMPC. The variation in the activation energy of medium-speed HW in DMPE compared with free HW in DMPC suggested the presence of various kinds of water networks around the headgroup.

## Data Availability

The data that support the findings of this study are available from the corresponding authors upon reasonable request.

## References

[1] J. Pieper , T. Hauss , A. Buchsteiner , K. Baczyński , K. Adamiak , R. E. Lechner , and G. Renger , “ Temperature- and hydration-dependent protein dynamics in photosystem II of green plants studied by quasielastic neutron scattering,” Biochemistry 46, 11398 (2007). 10.1021/bi700179s 17867656

[2] M. P. M. Marques , A. L. M. B. de Carvalho , A. P. Mamede , I. P. Santos , V. G. Sakai , A. Dopplapudi , G. Cinque , M. Wolna , P. Gardner , and L. A. E. B. de Carvalho , “ Chemotherapeutic targets in osteosarcoma: Insights from synchrotron-microFTIR and quasi-elastic neutron scattering,” J. Phys. Chem. B 123, 6968 (2019). 10.1021/acs.jpcb.9b05596 31339317

[3] M. Trapp , T. Gutberlet , F. Juranyi , T. Unruh , B. Demé , M. Tehei , and J. Peters , “ Hydration dependent studies of highly aligned multilayer lipid membranes by neutron scattering,” J. Chem. Phys. 133, 164505 (2010). 10.1063/1.3495973 21033803

[4] H. Aoki and M. Kodama , “ Calorimetric investigation of the behavior of interlamellar water in phospholipid-water system,” Thermochim. Acta 308, 77 (1998). 10.1016/S0040-6031(97)00334-1

[5] W. Zhao , D. E. Moilanen , E. E. Fenn , and M. D. Fayer , “ Water at the surfaces of aligned phospholipid multibilayer model membranes probed with ultrafast vibrational spectroscopy,” J. Am. Chem. Soc. 130, 13927 (2008). 10.1021/ja803252y 18823116 PMC2648527

[6] S. J. Marrink , M. Berkowitz , and H. J. C. Berendsen , “ Molecular dynamics simulation of a membrane/water interface: The ordering of water and its relation to the hydration force,” Langmuir 9, 3122 (1993). 10.1021/la00035a062

[7] R. E. Lechner , J. Fitter , N. A. Dencher , and T. Hau β, “ Dehydration of biological membranes by cooling: An investigation on the purple membrane,” J. Mol. Biol. 277, 593 (1998). 10.1006/jmbi.1997.1597 9533882

[8] A. Kundu , B. Blasiak , J. H. Lim , K. Kwak , and M. Cho , “ Water hydrogen-bonding network structure and dynamics at phospholipid multibilayer surface: Femtosecond mid-IR pump–probe spectroscopy,” J. Phys. Chem. Lett. 7, 741 (2016). 10.1021/acs.jpclett.6b00022 26859047

[9] M. Hishida and K. Tanaka , “ Long-range hydration effect of lipid membrane studied by terahertz time-domain spectroscopy,” Phys. Rev. Lett. 106, 158102 (2011). 10.1103/PhysRevLett.106.158102 21568617

[10] M. Hishida , K. Tanaka , Y. Yamamura , and K. Saito , “ Cooperativity between water and lipids in lamellar to inverted-hexagonal phase transition,” J. Phys. Soc. Jpn. 83, 044801 (2014). 10.7566/JPSJ.83.044801

[11] T. Yamada and H. Seto , “ Quasi-elastic neutron scattering studies on hydration water in phospholipid membranes,” Front. Chem. 8, 8 (2020). 10.3389/fchem.2020.00008 32039163 PMC6993101

[12] V. F. Sears , *Thermal-Neutron Scattering Lengths and Cross Sections for Condensed-Matter Research* ( Atomic Energy of Canada Ltd, Chalk River, Ontario, Canada, 1984).

[13] T. Yamada , N. Takahashi , T. Tominaga , S. Takata , and H. Seto , “ Dynamical behavior of hydration water molecules between phospholipid membranes,” J. Phys. Chem. B 121, 8322 (2017). 10.1021/acs.jpcb.7b01276 28787155

[14] S. König , E. Sackmann , D. Richter , R. Zorn , C. Carlile , and T. M. Bayerl , “ Molecular dynamics of water in oriented DPPC multilayers studied by quasielastic neutron scattering and deuterium-nuclear magnetic resonance relaxation,” J. Chem. Phys. 100, 3307 (1994). 10.1063/1.466422

[15] V. K. Sharma , E. Mamontov , D. B. Anunciado , H. O'Neill , and V. Urban , “ Nanoscopic dynamics of phospholipid in unilamellar vesicles: Effect of gel to fluid phase transition,” J. Phys. Chem. B 119, 4460 (2015). 10.1021/acs.jpcb.5b00220 25738532

[16] L. Toppozini , F. Roosen-Runge , R. I. Bewley , R. M. Dalgliesh , T. Perring , T. Seydel , H. R. Glyde , V. G. Sakai , and M. C. Rheinstädter , “ Anomalous and anisotropic nanoscale diffusion of hydration water molecules in fluid lipid membranes,” Soft Matter 11, 8354 (2015). 10.1039/C5SM01713K 26338138

[17] J. Swenson , F. Kargl , P. Berntsen , and C. Svanberg , “ Solvent and lipid dynamics of hydrated lipid bilayers by incoherent quasielastic neutron scattering,” J. Chem. Phys. 129, 045101 (2008). 10.1063/1.2955753 18681680

[18] S. Busch , C. Smuda , L. C. Pardo , and T. Unruh , “ Molecular mechanism of long-range diffusion in phospholipid membranes studied by quasielastic neutron scattering,” J. Am. Chem. Soc. 132, 3232 (2010). 10.1021/ja907581s 20163140

[19] G. Schirò , Y. Fichou , F. X. Gallat , K. Wood , F. Gabel , M. Moulin , M. Härtlein , M. Heyden , J.-P. Colletier , A. Orecchini , A. Paciaroni , J. Wuttke , D. J. Tobias , and M. Weik , “ Translational diffusion of hydration water correlates with functional motions in folded and intrinsically disordered proteins,” Nat. Commun. 6, 6490 (2015). 10.1038/ncomms7490 25774711 PMC4382692

[20] H. Seto and T. Yamada , “ Quasi-elastic neutron scattering study of the effects of metal cations on the hydration water between phospholipid bilayers,” Appl. Phys. Lett. 116, 133701 (2020). 10.1063/1.5144012

[21] Y. Higuchi , Y. Asano , T. Kuwahara , and M. Hishida , “ Rotational dynamics of water at the phospholipid bilayer depending on the head groups studied by molecular dynamics simulations,” Langmuir 37, 5329 (2021). 10.1021/acs.langmuir.1c00417 33881324

[22] M. Hishida , A. Endo , K. Nakazawa , Y. Yamamura , and K. Saito , “ Effect of *n*-alkanes on lipid bilayers depending on headgroups,” Chem. Phys. Lip. 188, 61 (2015). 10.1016/j.chemphyslip.2015.05.002 25957868

[23] X. Wang , H. Takahashi , I. Hatta , and P. J. Quinn , “ An x-ray diffraction study of the effect of α-tocopherol on the structure and phase behaviour of bilayers of dimyristoylphosphatidylethanolamine,” Biochim. Biophys. Acta 1418, 335 (1999). 10.1016/S0005-2736(99)00044-9 10320684

[24] K. Shibata , N. Takahashi , Y. Kawakita , M. Matsuura , T. Yamada , T. Tominaga , W. Kambara , M. Kobayashi , Y. Inamura , T. Nakatani , K. Nakajima , and M. Arai , “ The performance of TOF near backscattering spectrometer DNA in MLF, J–PARC,” JPS Conf. Proc. 8, 036022 (2015). 10.7566/JPSCP.8.036022

[25] H. Seto , S. Itoh , T. Yokoo , H. Endo , K. Nakajima , K. Shibata , R. Kajimoto , S. Ohira-Kawamura , M. Nakamura , Y. Kawakita , H. Nakagawa , and T. Yamada , “ Inelastic and quasi-elastic neutron scattering spectrometers in J-PARC,” Biochim. Biophys. Acta, Gen. Subj. 1861, 3651 (2017). 10.1016/j.bbagen.2016.04.025 27156489

[26] M. J. Abraham , T. Murtola , R. Schulz , S. Páll , J. C. Smith , B. Hess , and E. Lindahl , “ GROMACS: High performance molecular simulations through multi-level parallelism from laptops to supercomputers,” SoftwareX 1–2, 19 (2015). 10.1016/j.softx.2015.06.001

[27] S. Páll , M. J. Abraham , C. Kutzner , B. Hess , and E. Lindahl , “ Tackling exascale software challenges in molecular dynamics simulations with GROMACS,” in *International Conference on Exascale Applications and Software*, edited by S. Markidis and E. Laure ( LNTCS, 2015), Vol. 8759.

[28] M. Parrinello and A. Rahman , “ Crystal structure and pair potentials: A molecular-dynamics study,” Phys. Rev. Lett. 45, 1196 (1980). 10.1103/PhysRevLett.45.1196

[29] S. Nosé , “ A unified formulation of the constant temperature molecular dynamics methods,” J. Chem. Phys. 81(1), 511 (1984). 10.1063/1.447334

[30] W. G. Hoover , “ Canonical dynamics: Equilibrium phase-space distributions,” Phys. Rev. A 31, 1695 (1985). 10.1103/PhysRevA.31.1695 9895674

[31] E. L. Wu , X. Cheng , S. Jo , H. Rui , K. C. Song , E. M. Dávila-Contreras , Y. Qi , J. Lee , V. Monje-Galvan , R. M. Venable , J. B. Klauda , and W. Im , “ CHARMM-GUI membrane builder toward realistic biological membrane simulations,” J. Comput. Chem. 35, 1997 (2014). 10.1002/jcc.23702 25130509 PMC4165794

[32] S. Jo , J. B. Lim , J. B. Klauda , and W. Im , “ CHARMM-GUI membrane builder for mixed bilayers and its application to yeast membranes,” Biophys. J. 97, 50 (2009). 10.1016/j.bpj.2009.04.013 19580743 PMC2711372

[33] S. Jo , T. Kim , and W. Im , “ Automated builder and database of protein/membrane complexes for molecular dynamics simulations,” PLoS One 2(9), 880 (2007). 10.1371/journal.pone.0000880 PMC1963319 17849009

[34] S. Jo , T. Kim , V. G. Iyer , and W. Im , “ CHARMM-GUI: A web-based graphical user interface for CHARMM,” J. Comput. Chem. 29, 1859 (2008). 10.1002/jcc.20945 18351591

[35] B. Hess , H. Bekker , H. J. C. Berendsen , and J. G. E. M. Fraaije , “ LINCS: A linear constraint solver for molecular simulations,” J. Comput. Chem. 18, 1463 (1997). 10.1002/(SICI)1096-987X(199709)18:12<1463::AID-JCC4>3.0.CO;2-H

[36] S. Miyamoto and P. A. Kollman , “ SETTLE: An analytical version of the SHAKE and RATTLE algorithms for rigid water models,” J. Comput. Chem. 13, 952 (1992). 10.1002/jcc.540130805

[37] U. Essmann , L. Perera , M. L. Berkowitz , T. Darden , H. Lee , and L. G. Pedersen , “ A smooth particle mesh Ewald method,” J. Chem. Phys. 103, 8577 (1995). 10.1063/1.470117

[38] M. Bee , *Quasielastic Neutron Scattering* ( Adam Hilger, Bristol, 1988).

[39] U. Wanderlingh , G. D'Angelo , C. Branca , V. C. Nibali , A. Trimarchi , S. Rifici , D. Finocchiaro , C. Crupi , J. Ollivier , and H. D. Middendorf , “ Multi-component modeling of quasielastic neutron scattering from phospholipid membranes,” J. Chem. Phys. 140, 174901 (2014). 10.1063/1.4872167 24811662

[40] C. L. Armstrong , M. D. Kaye , M. Zamponi , E. Mamontov , M. Tyagi , T. Jenkins , and M. C. Rheinstädter , “ Diffusion in single supported lipid bilayers studied by quasi-elastic neutron scattering,” Soft Matter 6, 5864 (2010). 10.1039/c0sm00637h

[41] C. L. Armstrong , M. Trapp , J. Peters , T. Seydel , and M. C. Rheinstädter , “ Short range ballistic motion in fluid lipid bilayers studied by quasi-elastic neutron scattering,” Soft Matter 7, 8358 (2011). 10.1039/c1sm05691c

[42] W. Pfeiffer , T. Henkel , E. Sackmann , W. Knoll , and D. Richter , “ Local dynamics of lipid bilayers studied by incoherent quasi-elastic neutron scattering,” Europhys. Lett. 8(2), 201 (1989). 10.1209/0295-5075/8/2/016

[43] T. Matsuo , A. Cisse , M. Plazanet , F. Natali , M. M. Koza , J. Ollivier , D. J. Bicout , and J. Peters , “ The dynamical Matryoshka model: 3. Diffusive nature of the atomic motions contained in a new dynamical model for deciphering local lipid dynamics,” Biochim. Biophys. Acta, Biomembr. 1864, 183949–182022 (2022). 10.1016/j.bbamem.2022.183949 35508224

[44] D.-H. Choi , H. Son , S. Jung , J. Park , W.-Y. Park , O. S. Kwon , and G.-S. Park , “ Dielectric relaxation change of water upon phase transition of a lipid bilayer probed by terahertz time domain spectroscopy,” J. Chem. Phys. 137, 175101 (2012). 10.1063/1.4764304 23145747

[45] K. Wood , M. Plazanet , F. Gabel , B. Kessler , D. Oesterhelt , D. J. Tobias , G. Zaccai , and M. Weik , “ Coupling of protein and hydration-water dynamics in biological membranes,” Proc. Natl. Acad. Sci. U. S. A. 104, 18049 (2007). 10.1073/pnas.0706566104 17986611 PMC2084294

[46] See the supplementary material (https://sd.peerx-press.org/ms_files/sd/2023/07/28/00001111/02/1111_2_data_set_32506_ryhq3l.pdf) for additional information, data, and figures, as mentioned in the text.

